# Adherence to the Mediterranean Diet Is Associated with Better Metabolic Features in Youths with Type 1 Diabetes

**DOI:** 10.3390/nu14030596

**Published:** 2022-01-29

**Authors:** Valentina Antoniotti, Daniele Spadaccini, Roberta Ricotti, Deborah Carrera, Silvia Savastio, Filipa Patricia Goncalves Correia, Marina Caputo, Erica Pozzi, Simonetta Bellone, Ivana Rabbone, Flavia Prodam

**Affiliations:** 1 SCDU of Pediatrics, Department of Health Sciences, University of Piemonte Orientale, 28100 Novara, Italy; valentina.antoniotti@uniupo.it (V.A.); roberta.ricotti@uniupo.it (R.R.); savastio.silvia@gmail.com (S.S.); ericapozzi@yahoo.it (E.P.); simonetta.bellone@med.uniupo.it (S.B.); ivana.rabbone@uniupo.it (I.R.); 2 Department of Health Sciences, University of Piemonte Orientale, 28100 Novara, Italy; daniele.spadaccini@uniupo.it (D.S.); filipa.p.g.correia@gmail.com (F.P.G.C.); marina.caputo@uniupo.it (M.C.); 3 Pediatric Endocrinology, Azienda Ospedaliero Universitaria Maggiore Della Carità, 28100 Novara, Italy; carrera.deborah@gmail.com; 4 Endocrinology, Department of Translational Medicine, University of Piemonte Orientale, 28100 Novara, Italy

**Keywords:** diabetes, Mediterranean diet, pediatrics, food, nutrition, glucose, weight, blood pressure

## Abstract

Our aim was to evaluate adherence to the Mediterranean diet (MedDiet) among children and adolescents with type 1 diabetes (T1D) in relation to metabolic control. Adherence to the MedDiet was assessed with the Mediterranean Diet Quality Index (KIDMED) questionnaire and physical activity by the International Physical Activity Questionnaire for Adolescent (IPAQ-A) on 65 subjects (32 males, 9–18 years) with T1D. Clinical and metabolic evaluation was performed (standardized body mass index (BMI-SDS), hemoglobin A1C (HbA1c), continuous glucose monitoring metrics when present, blood pressure, lipid profile). Parental characteristics (age, body mass index (BMI), socio-economic status) were reported. The adherence to the MedDiet was poor in 12.3%, average in 58.6%, and high in 29.1% of the subjects. Furthermore, 23.4% of patients were overweight/obese. The most impacting factors on BMI-SDS were skipping breakfast and their father’s BMI. HbA1c and time in range % were positively associated with sweets and fish intake, respectively. Additionally, the father’s socio-economic status (SES) and mother’s age were associated with glucose control. Blood pressure was associated with travelling to school in vehicles, extra-virgin olive oil intake and milk/dairy consumption at breakfast. The promotion of the MedDiet, mainly having a healthy breakfast, is a good strategy to include in the management of T1D to improve glucose and metabolic control. This research is valuable for parents to obtain the best results for their children with T1D.

## 1. Introduction

Type 1 diabetes (T1D) is an autoimmune disease that is rapidly increasing worldwide [1]. It is also one of the most common chronic diseases in childhood with an estimated prevalence of 1.1 million children and adolescents affected [2]. As well as insulin treatment, a crucial part of T1D patient therapy [3,4,5], another treatment is medical nutritional therapy (MNT). Its role is known in the management of type 2 diabetes, but it is also essential for T1D because of the need to adapt insulin therapy to nutrient intake, ensuring satisfactory glucose control [3,5,6]. Increased nutritional awareness of patients and their families is associated with better glucose control and the high quality of the diet in young people [7]. MNT assigned by a dietitian or a registered nutritionist is associated with a 1.0–1.9% decrease in Hba1c for people with T1D [8].

MNT includes recommendations for healthy eating patterns since patients with T1D are at high cardiovascular risk. The 2018 International Society for Pediatric and Adolescent Diabetes (ISPAD) Guidelines recommend as proper nutrition habits for diabetes in children, a diet that allows for optimal growth, ideal weight maintenance and the prevention of acute and chronic complications of diabetes mellitus. The approximate energy intake and the essential nutrients should be distributed as follows: carbohydrates 45–55%, fats 30–35% and proteins 15–20% [5]. The bromatological composition is in line with that of the Mediterranean diet (MedDiet). In addition, there is evidence that low-carbohydrate diets can be nutritionally inadequate and may increase the risk of hypoglycemia [9]. Eating patterns like the MedDiet (based on wholegrain cereals, monounsaturated fats, plant-based food, reduced intake of red and processed meats) were suggested to be beneficial for long-term health, reducing cardiovascular risk [3,10]. The relationship between the MedDiet and diabetes mellitus has been widely studied, specifically related to type 2 diabetes mellitus (T2D) in adults, documenting beneficial effects in patients in terms of glucose control and primary and secondary prevention [11,12]. Several observational and some interventional studies demonstrated beneficial effects of the MedDiet on glucose homeostasis in individuals with T2D or metabolic syndrome. In a cross-sectional study involving 901 patients with T2D, the largest degree of adherence to the MedDiet was associated with low levels of hemoglobin A1C (HbA1c) and better post-prandial blood glucose control [13]. In addition to T2D, a multicenter observational study of 1076 pregnant women from ten Mediterranean countries, supported a reverse association between adherence to the MedDiet and the likelihood of gestational diabetes mellitus [14].

Data on nutritional habits in youths with T1D are scarce. Few studies have evaluated the effectiveness of the MedDiet in improving glucose control and the lipid profile. The main studies regarding T1D involved adult subjects. Observational data of the collaboration of European Childhood Diabetes Registers (EURODIAB) Prospective Complications Study examined the effects of saturated fatty acid (SFA) and dietary fibers on the onset of cardiovascular disease, showing an inverse correlation between fiber intake and cardiovascular risk in these patients [15]. Furthermore, the SEARCH Nutrition Ancillary Study confirmed that high adherence to the MedDiet was associated, overtime, with low levels of HbA1c and lipids in US youths [16]. These findings agree with an intervention study on a structured education about the MedDiet conducted in Italy by our group on adolescents with T1D [10]. The adherence to the MedDiet seemed more favorable than that to the Dietary Approaches to Stop Hypertension (DASH) diet since its impact was demonstrated only in a cross-sectional study [16,17].

In the context of the limited reported data, the purpose of this study was to assess the nutritional habits and physical activity in a group of multi-ethnic youths with T1D in North of Italy, surveyed in relation to their adherence to the MedDiet and metabolic parameters.

## 2. Subjects and Methods

### 2.1. Population and Clinical Parameters

The subjects were 65 consecutive youths aged between 9 and 18 years old recruited from outpatients attending the Division of Pediatrics of our Hospital between 2018 and 2021. The study was approved by the Ethical Committee of Novara (protocol number 143/17) and conformed to the guidelines of the European Convention of Human Rights and Biomedicine for Research in Children. Patients were diagnosed with T1D according to the American Diabetes Association (ADA) criteria [3]. We included multi-ethnic subjects with a T1D diagnosis between 2002 and 2020 and a time from the onset of at least six months. Any HbA1c levels, insulin treatment (continuous insulin or multiple daily injections) and body mass index (BMI) were considered. Inability to understand the Italian language or difficult engagement with the cultural mediator was the only exclusion criteria. Weight, height and blood pressure (BP) were measured by the medical staff. The BMI and BMI z-score were calculated. Weight was measured to the nearest 100 g by using an electronic scale, and height by a Harpenden stadiometer to the nearest mm. BMI was calculated as the ratio between weight (kg) and squared height (m^2^). Normal weight, overweight and obesity were classified according to the International Obesity Task Force (IOTF) growth charts [18]. Blood pressure was evaluated as suggested by the National High Blood Pressure Educational Program (NHBPEP) Working Group of the American Academy of Pediatrics (AAP) [19]. Socio-economic status (SES) was assessed on the educational level of both parents and the type of work, classified as low, medium and high SES.

### 2.2. Dietary and Physical Activity Assessment

Adherence to the MedDiet was evaluated using the validated Italian version of the Mediterranean Diet Quality Index (KIDMED) score (Supplementary Table S1), which is composed of 16 dichotomous (positive/negative) items on eating habits [20]. The test is divided into four questions with negative connotations (−1) and twelve questions evaluated with a positive score (+1). A total score was calculated, ranging from 4 to 12. The assessment of the test was interpreted according to the following classification: low adherence (total score ≤ 3), average adherence (total score between 4 and 7), high adherence (total score ≥ 8). Items and scores are reported in Supplementary Table S1. The Italian version of the International Physical Activity Questionnaire for Adolescents (IPAQ-A) was used to assess the level of physical activity. It consists of four domains of physical activity reflecting on the activities of the previous seven days: school-related physical activity (including activities during physical education lessons and breaks), transport, housework and leisure physical activity [21]. The level of physical activity was measured by the Metabolic Equivalent of Task (MET). Both questionnaires were completed with a driven interview conducted by researchers who also collected anthropometric and social data of parents/caregivers.

### 2.3. Biochemical Evaluation and Glucose Monitoring Parameters

We collected biochemical data and glucose monitoring parameters of the routine clinical care. After overnight fasting, high-density lipoprotein (HDL), low-density lipoprotein(LDL), triglycerides, total cholesterol, glucose and HbA1c levels were measured. Daily insulin demand was calculated as both total IU/day and per kg/day. In 31 patients, additional values from a continuous glucose monitoring device (CGM) were also available. Outcomes of CGM were extracted, including average blood glucose (mg/dL) and standard deviation (SD), time in range (TIR) of optimal target (70–180 mg/dL) and variability classified as low (<70 mg/dL) and very low (<54 mg/dL), high (>180 mg/dL) and very high (>250 mg/dL). We considered as glucose imbalance Hba1c more than 8.5% and TIR lower than 70%, two validated cut-offs that represented approximately one-third of each group [22].

### 2.4. Statistical Analysis

All analyses were performed using the Statistical Package for the Social Sciences, Version 26.0 (SPSS Inc., Chicago, IL, USA). Descriptive characteristics were presented for each gender and full sample. After verification of the normal distribution of continuous variables through the Shapiro–Wilks test and PP plot (probability–probability plot), descriptive characteristics were shown as the mean + standard deviation, while qualitative variables were presented as frequencies or medians. The chi-squared test, the t-test for independent variables (means) and Mann–Whitney U-test (medians) were performed to study differences between genders and *p*-value was assessed for all tests. Correlation analysis followed the assumptions of Pearson *r* (parametric data) or Spearman’s *ρ* (non-parametric data) depending on the characteristics of the correlated variables. Statistically significant correlations were expressed as *r*/*ρ* and *p*-value < 0.05 or < 0.01. Regression analysis of quantitative continuous dependent variables was conducted for a restricted portion of studied variables by creating regression models through a stepwise linear regression method. Independent variables were chosen from statistically significant correlated variables and by excluding multicollinearity factors or contributing factors. The qualitative regression of ordinal data was performed through multinomial logistic analysis instead. Best fitting model/models were determined automatically from the software. The confidence interval (CI) was set at 95%.

## 3. Results

The clinical data of the enrolled patients are shown in Table 1. All the 65 recruited patients (age: 15.1 ± 2.3 years) completed the questionnaires. Most of the patients were normal weight (71.9%), with a prevalence of overweight and obesity of 15.6% and 7.8%, respectively. All patients were in insulin treatment by the time of the visit, with 69% using the basal-bolus treatment and the remaining 31% using subcutaneous infusion. Regarding the management of the disease, only 21.9% of subjects were able to maintain a TIR% over 70%, and HbA1c was >8.5% in 26.2% of subjects. Data were also analyzed according to gender (32 males, 33 females). Males were taller than females and had also lower weight z score and standardized body mass index (BMI SDS). Females were more overweight and obese than males nearly to significance. Metabolic characteristics were similar between genders.

The KIDMED average (median) score was lower than recommended in both genders, with an almost statistically significant difference between boys and girls (Δ = 1.15 pts, *p* = 0.082) (Table 1). KIDMED scores showed that (≥8) 29.1% of the subjects adhered to an optimal Mediterranean diet, (4–7) 58.5%of the subjects needed to improve, and (≤3) 12.3% of the whole sample exhibited a low-quality diet (Table 2). Concerning KIDMED items, frequencies and statistics for both genders are shown in Table 2. More than 80% of the subjects were eating at least one portion of vegetables each day, and more than 60% were eating at least two portions. Adequate fish and legume consumption was found in approximately 50% of the subjects. Males were more likely to eat at least one fruit/day (*p* < 0.01), ate less processed food at breakfast (*p* < 0.05) and more dairy foods daily (*p* < 0.05) compared to females.

Other characteristics of the sample, including the socio-economic aspects of families and the main physical activity items assessed through IPAQ-A, are presented in Table 3. The IPAQ-A additional items’ descriptive characteristics are shown in Supplementary Table S2. According to our data, mild physical activity was slightly more appreciated by the females, while vigorous physical activity was higher in males, even if the difference was not statistically significant (*p* = ns). Family count varied from two to six individuals with four individuals as the most frequent case (45.9%). The SES of the mother and father were not different, neither was the smoking habits of parents even if the fathers had twice the frequency of smoking (29.5%) compared to the mothers (14.8%).

### 3.1. Correlations and Regressions

#### 3.1.1. Weight and BMI

The evaluation of significant correlations between organic/anthropometric parameters and non-biological/nutritional/physical activity habits has provided a lot of data which are summarized in Supplementary Tables S3–S5. The weight z-score was correlated with a higher family count (*ρ* = 0.321; *p* < 0.05), skipping breakfast (*ρ* = −0.407; *p* = 0.001) and with the weight and BMI of parents. BMI-SDS kept the same tendency but added a direct correlation with legume consumption (*ρ* = 0.262 < 0.05). Increased IOTF-BMI was also correlated with a reduced intake of nuts (*ρ* = −0.425; *p* < 0.001), cereals (*ρ* = −0.248, <0.05), dairy foods at breakfast (*ρ* = −0.306; *p* < 0.05) and lower 8 MET activities (−0.249; *p* < 0.05). Stepwise regression (Table 4) showed that, for the whole sample, the most impacting independent factor on BMI-SDS was skipping breakfast, followed by the father’s BMI. Considering the differences between genders, BMI-SDS was primarily associated with skipping breakfast and father’s weight in females, while in males the main predicting factor was the time spent in physical activity during school even if skipping breakfast, reduced nut consumption and a higher family count (*p* < 0.05) were found to be correlated with increased BMI-SDS in males. IOTF-BMI class was primarily associated with nut consumption and 8 MET above all other effects.

#### 3.1.2. Glucose Control

Mean blood glucose was correlated with a lower age of the mother (*r* = −0.480; *p* < 0.01), the SES of father (*ρ* = −0.516; *p* < 0.01), fish consumption (*ρ* = −0.269; *p* < 0.05) and cereal consumption at breakfast (*ρ* = −0.418; *p* < 0.05). No aspects of physical activity were found to influence mean glucose. Beyond the correlations found for fasting blood glucose, HbA1c was also correlated with a higher consumption of sweets (*ρ* = −0.347; *p* < 0.01) and less days and time spent on a bike (*ρ* = −0.266 and *ρ* = −0.276 respectively; *p* < 0.05).TIR%, which was highly and inversely correlated with HbA1c (*ρ* = −0.881; *p* < 0.001), as expected, kept almost the same significant correlations of the latter: higher fish consumption (*ρ* = 0.390; *p* < 0.05) and cereals at breakfast (*ρ* = 0.427; *p* < 0.05), higher age of the mother (*r* = 0.401; *p* < 0.05), and the SES of the father (*ρ* = 0.471; *p* < 0.01).Stepwise regression (Table 5) conducted on these three main indicators of glucose control revealed that mean blood glucose was primarily predicted by the age of the mother and the SES of the father, while HbA1c was mainly associated with the SES of the father and the consumption of sweets. TIR% was instead even more associated with fish consumption, and its relationship with the SES of the father was revealed to be multiplicatively and independently associated with TIR% as the first model of regression. The KIDMED score was found only inversely correlated with the risk of hyperglycemia (*ρ* = 0.387; *p* < 0.05).

#### 3.1.3. Blood Pressure and Others

Blood pressure was found to be mainly linked with breakfast habits. Diastolic blood pressure (DBP) was correlated with less cereal consumption in the morning (*ρ* = −0.264; *p* < 0.05) and the consequently higher processed products consumed at breakfast (*ρ* = 0.260; *p* < 0.05). In addition, DPB was inversely correlated with extra-virgin olive oil intake (*ρ* = −0.296; *p* < 0.05). Systolic blood pressure (SPB) correlated with less milk/dairy foods at breakfast instead (*ρ* = −0.291; *p* < 0.05) and almost significantly with extra virgin olive oil consumption (*p* < 0.057). Physical activity items were found to be partially correlated with blood pressure, in particular traveling by car or public transport was correlated with both DBP (*ρ* = 0.338; *p* < 0.01) and SBP (*ρ* = 0.301; *p* < 0.01). Subsequent stepwise regression analysis (Table 6) confirmed that DBP was firstly associated with motorized travels for reaching school, followed by the other factors, while SPB was mainly predicted by olive oil and milk/dairy consumption at breakfast. Correlation analysis also showed that blood lipids were not linked to any of the nutritional and physical activity items (*p* = ns).

## 4. Discussion

MNT is a crucial element to improve glucose control and reduce cardiovascular risk in T1D in youths. Among dietary patterns, increasing evidence supports the MedDiet, mainly in adults with T2D [23], due its antioxidant and anti-inflammatory properties; however, results in the pediatric age should be implemented. This is one of the few studies that has aimed to describe the relationship among the components of the MedDiet and global metabolic control in children and adolescents with T1D.

### 4.1. Dietary Factors Associated with the Risk of Obesity

We observed that the prevalence of youths with T1D being overweight and obese is similar to the healthy pediatric population living in Italy [24,25,26] who also follow as an average adherence to the MedDiet [27,28]. Similar findings on the weight status of children with T1D have been reported in other ethnic groups or countries corroborating the general global trend [29,30,31,32,33,34]. Furthermore, females in our cohort were more at risk similar to other reports [32,33,35]. In all these studies, several factors have been associated with this risk of overweight/obesity in T1D as increased functional growth hormone (GH) secretion, longer disease duration, intensive insulin therapy in relation to pubertal insulin-resistance or flexible eating patterns, high insulin doses that could inhibit protein catabolism and slow basal metabolism or frequent snacking to avoid hypoglycemia [36,37]. However, only a few authors focused on dietary risk factors and even fewer on the MedDiet. Two US studies conducted using 287 pediatric subjects aged between 8 and 21 years old showed that the obesity risk was related to a frequent intake of unhealthy foods poor in fibers and micronutrients [38,39]. Interestingly, we observed that increased weight is associated with breakfast skipping habits, confirmed by the general pediatric population, as recently reviewed by our group in both cross-sectional and intervention trials [40,41]. The effect of skipping the first morning meal, despite being made up by similar calories in the day, is complex and partially unexplained, although several mechanisms have been hypothesized, such as circadian misalignment, the length of the night fasting and other unhealthy food habits. However, the confirmation in children and adolescents with T1D suggests that the alteration of the chronotype is a key feature of obesity development in any condition. Furthermore, we observed that the intake of dairy products at breakfast was also negatively associated with the risk of being overweight and obese. With the urbanization of people living in the Mediterranean area, youth are deviating to a Western diet rich in saturated fat, simple carbohydrates and refined and processed foods. One of the typical phenomena of this nutrition transition is the choice of sugar-sweetened beverages at breakfast instead of dairy products [42]. Other studies have recently reported this association in children with obesity [43,44,45,46,47]. Behind the excess of simple sugars instead of foods rich in nutrients, several other mechanisms have been reported, such as derangements in the calcium homeostasis, insulin secretion, satiety and satiation and gut-microbiota when the intake of dairy products is low [45,48,49]. In our cohort a low intake of nuts and cereals was associated with an increased risk of being overweight and obese. Two studies on adults with T1D, the Finnish Diabetic Nephropathy (FinnDiane) Study of 1058 individuals [50] and the EURODIAB Complications Study of 2868 individuals [51], confirmed our pediatric results on nuts and cereals, respectively. These findings could hide a high consumption of unhealthy processed foods rich in saturated and trans fats, and sugars instead of nuts and cereals. Although nuts are dense-energy foods, they are rich in micronutrients and bio-active compounds. Growing evidence suggests their role in protecting from obesity or helping in weight loss [52,53,54] due to several mechanisms including prolonged chewing, delayed gastric emptying because of fibers and unsaturated fats, increased satiety and booster actions on lipid oxidation and thermogenesis [55]. 

### 4.2. Dietary Factors Associated with the Glucose Control

Although a better KIDMED score only reduced the risk of hyperglycemia differently from a modified version that correlated with HbA1c [16], low mean glucose levels, HbA1c and high TIR% were all associated with determinants of the KIDMED score, such as a high consumption of fish, cereals at breakfast, and a low consumption of sweets and candies. As previously discussed for weight, foods rich in nutrients and maintenance of breakfast habits are confirmed as being correlated with better glucose control, diversely from foods rich in saturated fats and sugars. Another study previously discussed on weight, observed that poor diabetic control was associated with unhealthy foods [39], as well as another one in Brazilian children with ultra-processed foods, a category that also comprises sweets and candies [56]. Although fasting glucose levels, HbA1c and TIR% were all correlated and associated with the same factors, interestingly, the main dietary determinants in the regression models were different. A high HbA1c was mostly related to a high intake of sweets which are rich in sugars. This is consistent with previous data on the fact that apart from total carbohydrate daily content, source of carbohydrate intake, and, therefore, glycemic load/index, is associated with HbA1c levels [57,58,59,60]. Furthermore, we observed that a high TIR was associated with a high intake of fish. No similar results have been published yet, whereas a better TIR has been recently associated with a carbohydrate intake range from 40–44% [61], or fish intake with a lower risk of microalbuminuria [62]. Fish is rich in macronutrients and micronutrients which are implicated with better metabolic control as well as low insulin resistance [63]. However, the human diet is complex, and different nutrients could have a synergistic effect. Because concluding data on the specific role of omega 3, vitamin D, unsaturated fats and proteins alone are still not conclusive concerning glucose control, and even less on TIR [64,65,66], further studies or post-hoc analysis from registries on T1D are needed.

### 4.3. Dietary Factors Associated with Blood Pressure

In line with findings on glucose controls, blood pressure was associated with breakfast habits. The high consumption of cereals and milk/dairy foods, and low consumption of processed products at breakfast were associated with better DBP and SBP levels.All these components of breakfast mirror healthy habits balanced in nutrients and are typical not only of the MedDiet but also of the DASH diet [67,68]. Adherence to it has been demonstrated to reduce the risk of hypertension in the SEARCH for Diabetes in Youth Study [69]. The effect on blood pressure dueto the consumption of processed foods is likely due to several factors, but surely one of the most important factors is the contribution of increasedsaltintake [70]. Regarding milk products, the relatively high potassium content of milk and dairy products implies that increased intake of them may reduce blood pressure, as also observed with studies on the DASH diet or lacto-ovo vegetarian diet [49,67]. This seems more pronounced when dairy products are consumed instead of juice or sugar-sweetened beverages [49,71,72]. No reports are present for T1D, distinct from T2D or metabolic syndrome [67,73], and our data strengthen the evidence in other diseases. 

Extra virgin olive oil (EVOO) reduces cardiovascular risk in adults as demonstrated by numerous studies, including the Nurses’ Health Study II, the Health Professional’s Follow-up Study and the Prevention with Mediterranean Diet (PREDIMED) Study [74]. Furthermore, both experimental and human observational and intervention studies demonstrated EVOO has anti-hypertensive actions due to its chemical composition, mainly characterized by oleic acid, polyphenols and other antioxidant compounds [75]. Our data on EVOO and DBP are in line with a recent intervention trial in adults with T1D in which EVOO improved vascular function [76].

### 4.4. Family, Social Factors and Physical Activity

Interestingly, we observed that BMI-SDS and glucose control were associated directly with the BMI of the father, the latter indirectly with the SES of the father and the mother’s age. It is well known that weight in offspring is partly inherited by parents but also due to the family setting, in particular the SES. All of these are markers of a complex interplay among genetics and environment that have the most significant impact on children younger than ten years old. However, data on the role of paternal BMI and mothers’ age is scarce due to the lack of studies for these factors [77,78,79]. Indeed, our findings strengthen some data in these subjects with regards to T1D. Regarding the role of the family on offspring concerning glucose control, while SES is associated with clear evidence, parental characteristics need further study because the role they play is inconclusive [80]. However, regarding the mother’s crucial involvement in T1D management [80], we can speculate that maternal age could result in different maternal parenting styles and coping abilities [81,82]. We observed some associations among physical activity determinants and weight and diastolic blood pressure. Most of the results are related to sedentary behaviors, such as going to school by car, which likely reflects a generally reduced physical activity that could be underreported. Our results strengthen the importance of empowerment to improve physical activity in youths with T1D to reduce cardiovascular risk [5,83].

### 4.5. Study Limitations and Strengths

This study has some limitations. First, it was a cross-sectional study design, and we could not establish causal relationships between MedDiet adherence and health outcomes. Second, we used the KIDMED score without integrating it with a food frequency questionnaire. However, periodically, our patients carried out a dietary survey by dieticians for the improvement of carbohydrate counting, indeed, they were educated about the assessment of diet quality. Furthermore, the KIDMED score is the most used index of adherence in the pediatric literature and by using it in the past we have obtained interesting results, and it has been validated through use on the general population [27,28]. Indeed, we cannot exclude further nutrients from being players behind the food habits we reported in T1D.Unexpectedly, we failed to show any correlation with fruit or vegetable intake. This could result from high consumption of fruits and vegetables in our cohort, likely due to the dietary counseling for T1D. Furthermore, physical activity had minimal impact on the features we analyzed. Although the International Physical Activity Questionnaire (IPAQ) was validated on children and adolescents [21], movement activities linked to free play among peers could be underreported. In general, we demonstrated that good adherence to the MedDiet, in particular to some food habits, was associated with a better metabolic status in youth with T1D. 

## 5. Conclusions

Dietary risk factors, typical of low adherence to the MedDiet, were associated with a high risk of obesity in T1D, which were the same as the general pediatric and adult population. This suggests that youths with T1D are not protected from increased weight whether they continuously adhere to healthy food patterns or not. The habit of having breakfast with foods rich in nutrients, including dairy products, is one of the most important dietary features we found associated with good glucose and metabolic control. The promotion of breakfast, as well as discouraging the excessive intake of ultra-processed foods, are confirmed key determinants in the prevention of negative health effects of nutrition transition in youth with T1D. Even if the cardiovascular protective role of EVOO is reaching amassing evidence in children, more attention and further research are needed to understand the role of fish consumption in glucose regulation. The promotion of the MedDiet is a good strategy to include in the management of youth with T1D, and all parents should be aware of this to obtain the best results for their children in the management of the disease.

## Figures and Tables

**Table 1:** Descriptive characteristics of sample.

		Full Sample	Boys	Girls	*p*-Value
Age (years)		15.07 ± 2.27	14.96 ± 2.07	15.18 ± 2.48	0.704
Years since T1D diagnosis (y)		6.15 ± 4.29	6.29 ± 4.64	6.03 ± 3.99	0.818
Weight (kg)		56.2 ± 13.22	56.71 ± 14.01	55.71 ± 12.61	0.762
Weight z-score (sd)		**−0.07 ± 1.16**	**−0.37 ± 1.17**	**0.22 ± 1.08**	**0.038**
Height (m)		**1.63 ± 0.11**	**1.66 ± 0.11**	**1.60 ± 0.09**	**0.025**
Height z-score (ds)		−0.00 ± 0.95	−0.12 ± 1.04	0.12 ± 0.85	0.319
BMI (kg/m^2^)		21.08 ± 3.85	20.37 ± 3.88	21.80 ± 3.74	0.140
BMI-SDS (sd)		**−0.13 ± 1.20**	**−0.44 ± 1.23**	**0.18 ± 1.10**	**0.036**
BMI-IOTF	−2 (extremely underweight)	1 (1.6%)	1 (3.1%)	0	0.060 ^1^
	−1 (underweight)	2 (3.1%)	2 (6.3%)	0	
	0 (normal weight)	46 (71.9%)	24 (75%)	22 (68.8%)	
	1 (overweight)	10 (15.6%)	3 (9.4%)	7 (21.9%)	
	2 (obese)	5 (7.8%)	2 (6.3%)	3 (9.4%)	
SBP (mmHg)		110.97 ± 10.10	110.48 ± 9.37	111.45 ± 10.91	0.226
DBP (mmHG)		69.34 ± 9.01	67.97 ± 8.69	70.71 ± 9.25	0.192
Mean Blood Glucose (mg/dL)		178.03 ± 32.25	180.80 ± 37.98	175.44 ± 26.80	0.651
HbA1c (%)		8.04 ± 1.78	8.16 ± 1.79	7.93 ± 1.79	0.609
TIR (%)		55.16 ± 18.54	53.42 ± 21.15	56.89 ± 16.02	0.604
UI insulin/kg/die		0.64 ± 0.26	0.64 ± 0.30	0.64 ± 0.23	0.894
Insulin treatment (1/2) ^2^		1 = 45 (69.2%) 2 = 20 (30.8%)	1= 23 (71.9%) 2 = 9 (28.1%)	1 = 22(66.6%) 2 = 11(33.3%)	0.644
Total Cholesterol (mg/dL)		162.21 ± 28.89	159.13 ± 32.02	165.19 ± 25.69	0.417
HDL Cholesterol (mg/dL)		59.77 ± 15.23	59.73 ± 15.99	59.81 ± 14.74	0.984
Triglycerides (mg/dL)		71.03 ± 42.72	74.67 ± 50.54	67.62 ± 34.31	0.521
KIDMED (pts)		6 (−1/+12)	6 (2/12)	6 (−1/+11)	0.082 ^1^

^1^: calculated with Mann–Whitney U-test. ^2^: 1—basal/bolus injection/2—intravenous infuser. Descriptive characteristics are expressed as mean ± ds or median (min/max) or as frequencies (percentages). *p*-value expresses difference between genders. Statistically significant differences in the variables between genders are showed in bold. T1D—Type 1 diabetes, BMI—Body Mass Index, BMI-SDS—Standardized Body Mass Index, BMI-IOTF—International Obesity Taskforce, SBP—systolic blood pressure, DPB—diastolic blood pressure, HbA1c—glycated hemoglobin, TIR—time in range, HDL—high density lipoprotein, KIDMED—Mediterranean Diet Quality Index.

**Table 2:** KIDMED items and other nutrition-related statistics in the sample.

		Full Sample	Boys	Girls	*p*-Value
**KIDMED class**	1	9 (13.8%)	2 (6.3%)	7 (21.2%)	0.55
	2	37 (56.9%)	18 (56.3%)	19 (57.6%)	
	3	19 (29.2%)	12 (37.5%)	7 (21.2%)	
Celiac disease (Y/N)		Y = 14 (21.5%); N = 51 (78.5%)	Y = 7 (21.9%); N = 25 (78.1%)	Y = 7 (21.2%); N = 26 (78.8%)	0.949
CHO calculation (Y/N)		Y = 44 (67.7%); N = 21 (32.2%)	Y = 22 (68.8%); N = 10 (31.3%)	Y = 22 (66.7%); N = 11 (33.3%)	0.859
1 p. Fruit/day (0/+1)		**0 = 14 (21.5%);** **1 = 51 (78.5%)**	**0 = 2 (6.3%);** **1 = 30 (93.8%)**	**0 = 12 (36.4%);** **1 = 21 (63.6%)**	**0.003**
2 p. Fruit/day (0/+1)		0 = 36 (55.4%); 1 = 29 (44.6%)	0 = 15 (46.9%); 1 = 17 (53.1%)	0 = 21 (63.6%); 1 = 12 (36.4%)	0.177
1 p. Vegetables/day (0/+1)		0 = 10 (15.4%); 1 = 55 (84.6%)	0 = 6 (18.8%); 1 = 26 (81.3%)	0 = 4 (12.1%); 1 = 29 (87.9%)	0.462
2 p. Vegetables/day (0/+1)		0 = 24 (36.9%); 1 = 41 (63.1%)	0 = 12 (37.5%); 1 = 20 (62.5%)	0 = 12 (36.4%); 1 = 21 (63.6%)	0.925
Fish/2 or 3 p. each week (0/+1)		0 = 29 (44.6%); 1 = 36 (55.4%)	0 = 14 (43.8%); 1 = 18 (56.3%)	0 = 15 (45.5%); 1 = 18 (54.5%)	0.891
Fast Food once a week (−1; 0)		−1 = 1 (1.5%); 0 = 64 (98.5%)	−1 = 1 (3.1%); 0 = 31 (96.9%)	0 = 33 (100%)	0.310
Legumes at least 1 p. a week (0; +1)		0 = 35 (53.8%); 1 = 30 (46.2%)	0 = 19 (59.4%); 1 = 13 (40.6%)	0 = 16 (48.5%); 1 = 17 (51.5%)	0.382
Cereals (pasta, rice…) at least 5 p. a week (0; +1)		0 = 2 (3.1%); 1 = 63 (96.9%)	1 = 32 (100%)	0 = 2 (6.1%); 1 = 31 (93.9%)	0.160
Cereals at breakfast (0; +1)		0 = 46 (70.8%); 1 = 19 (29.2%)	0 = 20 (62.5%); 1 = 12 (37.5%)	0 = 26 (78.8%); 1 = 7 (21.2%)	0.152
Nuts and similar foods 2 or 3 p. a week (0; +1)		0 = 48 (73.8%); 1 = 17 (26.2%)	0 = 21 (65.6%); 1 = 11 (34.4%)	0 = 27 (81.8%); 1 = 6 (18.2%)	0.141
Olive Oil as preferred oil (0; +1)		0 = 1 (1.5%); 1 = 64 (98.5%)	1 = 32 (100%)	0 = 1 (3%); 1 = 32 (97%)	0.325
Skip breakfast (−1; 0)		−1 = 7 (10.8%); 0 = 58 (89.2%)	−1 = 3 (9.4%); 0 = 29 (90.6%)	−1 = 4 (12.1%); 0 = 29 (87.9%)	0.723
Milk/yogurt or dairy food at breakfast (0; +1)		0 = 18 (27.7%); 1 = 47 (72.3%)	0 = 8 (25%); 1 = 24 (75%)	0 = 10 (30.3%); 1 = 23 (69.7%)	0.636
Processed Food at breakfast (−1; 0)		**−1 = 53 (81.5%);** **0 = 12 (18.5%)**	**−1 = 22 (68.8%);** **0 = 10 (31.3%)**	**−1 = 31 (93.9%);** **0 = 2 (6.1%)**	**0.034**
2 portions of milk/yogurt or dairy foods/day (0; +1)		**0 = 29 (44.6%);** **1 = 36 (55.4%)**	**0 = 10 (31.3%);** **1 = 22 (68.8%)**	**0 = 19 (57.6%);** **1 = 14 (42.4%)**	**0.034**
Sweets and/or candies every day (−1; 0)		−1 = 20 (30.8%); 0 = 45 (69.2%)	−1 = 11 (34.4%); 0 = 21 (65.6%)	−1 = 9 (27.3%); 0 = 24 (72.7%)	0.538

KIDMED class: 1—low-quality diet; 2 KIDMED class: 1—to improve; 3—optimal Mediterranean diet. (0/+1): 0—No, +1—Yes. (−1; 0): −1—Yes, 0—No. Statistically significant differences in the variables between genders are showed in bold.

**Table 3:** IPAQ-A global assessments and other non-nutritional aspects of sample.

		Full Sample	Boys	Girls	*p*-Value
Family count	2	2 (3.3%)	1 (3.6%)	1 (3%)	0.138
	3	13 (21.3%)	9 (32.1%)	4 (12.1%)	
	4	28 (45.9%)	11 (39.3%)	17 (51.5%)	
	5	12 (19.7%)	5 (17.9%)	7 (21.2%)	
	6	6 (9.8%)	2 (7.1%)	4 (12.1%)	
SES mother *	1	27 (42.2%)	13 (40.6%)	14 (43.8%)	0.623
	2	31 (48.4%)	15 (46.9%)	16 (50%)	
	3	6 (9.4%)	4 (12.5%)	2 (6.3%)	
SES father *	1	36 (56.3%)	19 (59.4%)	17 (53.1%)	0.564
	2	25 (39.1%)	12 (37.5%)	13 (40.6%)	
	3	3 (4.7%)	1 (3.1%)	2 (6.3%)	
Mother smoke (Y/N) **		Y = 9 (14.8%); N = 52 (85.2)%	Y = 5 (17.2%); N = 24 (82.9%)	Y = 4 (12.1%); N = 28 (84.8%)	0.602
Father smoke (Y/N) **		Y = 18 (29.5%); N = 43 (70.5%)	Y = 8 (27.6%); N = 21 (72.4%)	Y = 10 (31.3%); N = 22 (68.8%)	0.756
3.3 MET (kcal/week)		809.01 ± 844.37	687.33 ± 626.77	927.00 ± 1008.07	0.331
4 MET (kcal/week)		590.77 ± 760.65	654.38 ± 908.70	529.09 ± 591.00	0.535
8 MET (kcal/week)		817.23 ± 1045.97	975.00 ± 1105.19	664.24 ± 977.65	0.259
Total kcal burnt/week		2217.01 ± 1341.71	2316.70 ± 1272.58	2120.33 ± 1418.43	0.348

SES—Socio-economic status; 3.3 MET—kcal burnt with walking/mild physical activity in the last week; 4 MET—kcal burnt with moderate physical activity in the last week; 8 MET—kcal burnt with vigorous physical activity in the last week; *—difference between mother and father assessed through Χ^2^ test: *p* = 0.181; **—difference between mother and father assessed through Χ^2^ test: *p* = 0.063.

**Table 4:** Multiple stepwise regression analysis results of significant effects (independent variables) on BMI-SDS (dependent variable) and multiple logistic stepwise regression on IOTF-BMI.

Dependent Variables	Significant Effects	B (95% CI)	β	*p* Value
BMI-SDS (sd): Model 1	Skip breakfast (−1/0)	−1.829 (−2.746; −0.912)	−0.471	<0.001
BMI-SDS (sd): Model 2	Skip breakfast (−1/0)	−1.804 (−2.675; −0.932)	−0.464	<0.001
	Father’s BMI (kg/m^2^)	0.074 (0.018; 0.129)	0.299	0.01
IOTF-BMI (pts) *	Nut consumption (0/1)	/	/	0.001
	8 MET	/	/	<0.05
Boys: BMI-SDS (sd)	Minutes of sport at school (min)	−0.016 (−0.027; −0.006)	−0.549	0.003
Females: BMI-SDS (sd): Model 1	Father’s weight (kg)	0.033 (0.011; 0.054)	0.507	0.004
Females: BMI-SDS (sd): Model 2	Father’s weight (kg)	0.027 (0.007; 0.047)	0.424	0.007
	Skip breakfast (−1/0)	−1.377 (−2.459; −0.295)	−0.396	0.015

CI—confidence interval. *—difference between mother and father assessed through Χ^2^ test: *p* = 0.181.

**Table 5:** Multiple stepwise regression analysis results of significant effects (independent variables) on glucose control main parameters (dependent variables).

Dependent Variables	Significant Effects	B (95% CI)	β	*p* Value
Mean blood glucose (mg/dL) Model 1	Mother’s age (y)	−3.321 (−5.628; −1.015)	−0.480	0.006
Mean blood glucose (mg/dL) Model 2	Mother’s age (y)	−2.851 (−4.976; −0.727)	−0.412	0.010
	Father’s SES (1/2/3)	−20.834 (−36.655; −5.013)	−0.404	0.012
HbA1c (%) Model 1	Father’s SES (1/2/3)	−1.050 (−1.808; −0.291)	−0.345	0.008
HbA1c (%) Model 2	Father’s SES (1/2/3)	−1.106 (−1.817; −0.395)	−0.363	0.003
	Consumption of Sweets (−1/0)	−1.379 (−2.295; −0.464)	−0.352	0.004
TIR (%) Model 1	Father’s SES (1/2/3)	15.718 (6.230; 25.206)	0.564	0.002
TIR (%) Model 2	Father’s SES (1/2/3)	14.316 (5.403; 23.228)	0.513	0.003
	Fish consumption (0/1)	12.327 (1.014; 23.640)	0.348	<0.05

**Table 6:** Multiple stepwise regression analysis results of significant effects (independent variables) on blood pressure (dependent variables).

Dependent Variables	Significant Effects	B (95% CI)	β	*p* Value
SBP (mmHg) Model 1	Milk/Dairy food at breakfast (0/1)	−6.073 (−11.551; −0.595)	−0.275	0.030
SBP (mmHg) Model 2	Milk/Dairy food at breakfast (0/1)	−6.557 (−11.884; −1.230)	−0.297	0.017
	Olive oil (0/1)	−21.279 (−40.475; −2.083)	−0.268	0.030
DBP (mmHg) Model 1	Days to school with motorized transport (n)	1.764 (0.538; 2.991)	0.348	0.006
DBP (mmHg) Model 2	Days to school with motorized transport (n)	1.779 (0.601; 2.957)	0.351	0.004
	Cereals at breakfast (0/1)	−5.567 (−10.072; −1.061)	−0.287	0.016
DBP (mmHg) Model 3	Days to school with motorized transport (n)	1.560 (0.396; 2.724)	0.308	0.009
	Cereals at breakfast (0/1)	−7.005 (−11.592; 2.418)	−0.361	0.003
	Processed Food at breakfast (0/1)	5.738 (0.303; 11.173)	0.254	0.039
DBP (mmHg) Model 4	Days to school with motorized transport (n)	1.342 (0.194; 2.490)	0.265	0.023
	Cereals at breakfast (0/1)	−6.703 (−11.166; −2.241)	−0.346	0.004
	Processed Food at breakfast (0/1)	6.144 (0.854; 11.434)	0.272	0.024
	Olive oil (0/1)	−16.997 (−32.881; −1.114)	−0.240	0.036

## Data Availability

The data presented in this study are contained within the article and the Supplementary Materials.

## Supplementary Materials

The following are available online at https://www.mdpi.com/article/10.3390/nu14030596/s1, Table S1: KIDMED items, Table S2: Descriptive characteristics of specific IPAQ-A (Italian version) items in the sample, Table S3: Correlations between clinical characteristics and KIDMED items, Table S4: Correlations between clinical characteristics and IPAQ-A items, Table S5: Correlations between clinical characteristics of children and mother and father data.
