# Supplementary material for: Adherence to the Mediterranean Diet Is Associated with Better Metabolic Features in Youths with Type 1 Diabetes
Source: Nutrients. 2022 Jan 29;14(3):596. doi: 10.3390/nu14030596 (PMC8840273; doi:10.3390/nu14030596)
Supplement: Supplementary file 1 [file nutrients-14-00596-s001.zip › nutrients-1568947-supplementary.pdf]

Table S1. KIDMED items.[1]Items with positive answer (+1).[2]Items with a negative score (−1).

KIDMED Items
Consumption of a fruit or a fruit juice every day[1]
Consumption of a second fruit every day[1]
Consumption of raw or cooked vegetables 1 time a day[1]
Consumption of raw or cooked vegetables >1 time a day[1]
Consumption of fish regularly (at least 2–3 times a week)[1]
Eating >1 time per week to a fast-food (hamburger) restaurant[2]
Consumption of beans >1 time per week[1]
Consumption of pasta or rice almost every day (≥5 times a week)[1]
Consumption of cereals or grains (bread etc.) for breakfast[1]
Consumption of nuts regularly (at least 2-3 times per week)[1]
Consumption of olive oil at home[1]
Skipping breakfast[2]
Consumption of a dairy product for breakfast (yoghurts, milks etc)[1]
Consumption of commercially baked goods or pastries for breakfast[2]
Consumption of 2 yoghurts and/or cheese (40 g) daily[1]
Consumption of sweets or candy several times every day[2]

Table S2. Descriptive characteristics of specific IPAQ-A (Italian version) items in the sample.

		Full Sample	Males	Females	*p*-value
School related physical activity	n° of sports lessons	0 (15) 23.1%; 1 (36) 55.4%; 2 (13) 20%; 4 (1) 1.5%	0 (7) 21.9%; 1 (19) 59.4%; 2 (5) 15.6%; 4 (1) 3.1%	0 (8) 24.2%; 1 (17) 51.5%; 2 (8) 24.2%	0.901
	mins of walking during sports lessons	18.77 ± 24.70	19.69 ± 26.27	17.88 ± 23.45	0.770
	mins of sport during sports lessons	60.15 ± 44.76	66.56 ± 46.15	53.94 ± 43.17	0.259
	days vigorous physical activity during school	0 (63) 96.9%; 5 (2) 3.1%	0 (30) 93.8%; 5 (2) 6.3%	0 (33) 100%	0.148
	minutes of vigorous activity during school	0.92 ± 5.86	1.87 ± 8.30	0.00 ± 0.00	0.199
	days moderate physical activity during school	0 (63) 96.9%; 2 (1) 1.5%; 3 (1) 1.5%	0 (31) 96.9%; 2 (1) 3.1%	0 (32) 97%; 3 (1) 3%	1.000
	minutes of moderate activity during school	1.38 ±8.27	1.87 ± 10.61	0.91 ±5.22	0.641
	days of walking during school	0 (64) 98.5%; 5 (1) 1.5%	0 (31) 96.9%; 5 (1) 3.1%	0 (33) 100%	0.310
	minutes of walking during school	0.31 ± 2.48	0.63 ± 3.54	0.00 ± 0.00	0.314
Home related physical activity	days of moderate activity at home	0 (25) 38.5%; 1 (12) 18.5%; 2 (12) 18.5%; 3 (7) 10.8%; 5 (1) 1.5%; 6 (1) 1.5%; 7 (7) 10.8%	0 (15) 46.9%; 1 (5) 15.6%; 2 (5) 15.6%; 3 (4) 12.5%; 5 (1) 3.1%; 7 (2) 6.3%	0 (10) 30.3%; 1 (7) 21.2%; 2 (7) 21.2%; 3 (3) 9.1%; 6 (1) 3%; 7 (5) 15.2%	0.205
	minutes of moderate activity at home	29.54 ± 47.00	25.94 ± 52.39	33.03 ± 41.64	0.547
Physical activity during travels or moving to school	days travelling with motorized transport	0 (4) 6.2%; 1 (1) 1.5%; 2 (6) 9.2%; 3 (2) 3.1%; 5 (40) 61.5%; 6 (1) 1.5%; 7 (11) 16.9%	0 (3) 9.4%; 1 (1) 3.1%; 2 (4) 12.5%; 3 (1) 3.1%; 5 (18) 56.3%; 7 (5) 15.6%	0 (1) 3%; 2 (2) 6.1%; 3 (1) 3%; 5 (22) 66.7%; 6 (1) 3%; 7 (6) 18.2%	0.157
	minutes travelling with motorized transport	37.85 ± 37.11	43.75 ± 44.27	32.12 ± 28.06	0.209
	days travelling using bike	0 (56) 86.2%; 1 (2) 3.1%; 2 (3) 4.6%; 3 (2) 3.1%; 4 (1) 1.5%; 5 (1) 1.5%	0 (28) 87.5%; 1 (1) 3.1%; 2 (2) 6.3%; 4 (1) 3.1%	0 (28) 84.8%; 1 (1) 3%; 2 (1) 3%; 3 (2) 6.1%; 5 (1) 3%	0.718
	minutes travelling using bike	6.38 ± 19.91	9.38 ± 26.87	03.48 ± 08.70	0.236
	days of travelling by foot	0 (19) 29.2; 1 (5) 7.7; 2 (8) 12.3%; 3 (2) 3.1%; 4 (1) 1.5%; 5 (21) 32.3%; 7 (9) 13.8%	0 (11) 34.4%; 1 (1) 3.1%; 2 (4) 12.5%; 3 (1) 3.1%; 4 (1) 3.1%; 5 (11) 34.4%; 7 (3) 9.4%	0 (8) 24.2%; 1 (4) 12.1%; 2 (4) 12.1%; 3 (1) 3%; 5 (10) 30.3%; 7 (6) 18.2%	0.477
	minutes travelling by foot	25.78 ± 34.23	17.69 ± 20.85	33.64 ± 42.34	0.006
Other sources of physical activity	other days of vigorous activity	0 (34) 52.3%; 1 (7) 10.8%; 2 (12) 18.5%; 3 (8) 12.3%; 4 (4) 6.2%	0 (15) 46.9%; 1 (4) 12.5%; 2 (5) 15.6%; 3 (5) 15.6%; 4 (3) 9.4%	0 (19) 57.6%; 1 (3) 9.1%; 2 (7) 21.2%; 3 (3) 9.1%; 4 (1) 3%	0.292
	minutes of other days of vigorous activity	42.54 ± 48.89	47.81 ± 49.30	37.42 ± 48.70	0.396
	other days of moderate activity	0 (45) 69.2%; 1 (7) 10.8%; 2 (5) 7.7%; 3 (2) 3.1%; 4 (2) 3.1%; 5 (2) 3.1%; 6 (1) 1.5%; 7 (1) 1.5%	0 (21) 65.6%; 1 (4) 12.5%; 2 (3) 9.4%; 4 (2) 6.3%; 5 (1) 3.1%; 6 (1) 3.1%	0 (24) 72.7%; 1 (3) 9.1%; 2 (2) 6.1%; 3 (2) 6.1%; 5 (1) 3%; 7 (1) 3%	0.568

	**minutes other days of moderate activity**	26.54 ± 47.18	28.13 ± 50.19	25.00 ± 44.79	0.792
	**other days walking**	0 (54) 83.1%; 1 (2) 3.1%; 2 (3) 4.6%; 3 (3) 4.6%; 7 (3) 4.6%	0 (27) 84.4%; 1 (1) 3.1%; 2 (1) 3.1%; 3 (2) 6.3%; 7 (1) 3.1%	0 (27) 81.8%; 1 (1) 3%; 2 (2) 6.1%; 3 (1) 3%; 7 (2) 6.1%	0.778
	**minutes other days walking**	12.54 ± 31.67	11.25 ± 28.26	13.79 ± 35.07	0.750

Continuous variables (minutes of physical activity) are expressed as mean ± standard deviation while qualitative variables (days) are expressed as frequencies and percentages.

Table S3. Correlations between clinical characteristics and KIDMED items.

	KIDMED	KIDMED low-medium-high	Fruit 1	Fruit 2	Vegetable 1	Vegetable 2	Fish	Fast Food	Legumes	Pasta and cereals	Cereals at breakfast	Nuts	Extra virgin olive oil	Skip breakfast	Milk/dairy at breakfast	Processed food at breakfast	Dairy food 2/day	Sweets and candies
**Gender**	0.212	0.240	.366**	0.169	-0.092	-0.012	0.017	-0.127	-0.109	0.175	0.179	0.184	0.123	0.044	0.059	.325**	.265*	-0.077
**Age**	-0.001	0.011	-0.176	-0.172	-0.011	0.061	-0.067	-0.027	0.011	0.014	0.059	0.119	-0.193	-0.124	-0.218	0.114	.280*	-0.007
**Weight z-score**	0.046	-0.033	-0.068	0.035	0.091	0.202	-0.002	0.020	0.240	-0.014	-0.054	-0.114	-0.187	-.407**	0.000	-0.167	-0.178	0.062
**Height z-score**	0.078	0.087	0.032	0.103	-0.065	0.142	0.137	0.092	0.010	0.170	-0.035	0.073	-0.126	-0.005	-0.098	-0.144	0.017	0.012
**BMI**	-0.028	-0.112	-0.143	-0.095	0.119	0.145	-0.094	-0.038	.251*	-0.088	-0.058	-0.179	-0.188	-.405**	0.002	-0.134	-0.147	0.024
**BMI-SDS**	-0.030	-0.112	-0.106	-0.061	0.112	0.121	-0.080	-0.010	.264*	-0.092	-0.079	-0.198	-0.188	-.413**	0.021	-0.176	-0.196	0.069
**BMI IOTF**	-0.083	-0.151	-0.039	-0.055	0.147	0.172	-0.125	0.052	0.209	-0.098	-.248*	-.425**	-0.190	-.317*	0.012	-0.176	-.306*	0.048
**HbA1c**	-0.191	-0.209	0.019	-0.125	-0.209	-0.138	-.269*	-0.050	0.031	0.164	-0.060	-0.009	0.013	0.050	-0.022	-0.018	-0.013	-.347**
**SBP**	-0.058	-0.046	-0.021	-0.144	0.038	0.129	-0.039		0.130	-0.082	-0.147	-0.010	-0.216	0.037	-.291*	-0.005	0.007	-0.009
**DBP**	-0.033	-0.144	-0.100	-0.128	-0.062	0.191	0.088		0.149	-0.221	-.264*	-0.057	-0.211	-0.133	-0.168	0.191	-0.162	0.119
**Years from diagnosis**	0.004	-0.049	-0.138	-0.194	0.084	0.019	-0.121	0.003	0.027	-0.076	0.121	-0.007	-0.187	0.008	-0.048	0.179	0.095	-0.102
**UI/Kg/die**	-0.121	-0.109	0.034	-0.146	0.105	-0.199	-0.068	-0.020	0.188	0.128	-0.011	-0.069	-0.067	0.061	-0.082	-0.201	0.037	-0.077
**Glucose**	-0.236	-0.304	0.160	-0.308	-0.220	0.004	-.431*		0.062	0.257	-.418*	-0.033	-0.143	-0.046	0.096	-0.219	-0.076	-0.242
**TIR %**	0.230	0.258	-0.233	0.208	0.017	-0.053	.390*		-0.132	-0.238	.427*	0.141	0.146	0.156	-0.117	0.270	0.075	0.172
**TC**	0.039	0.003	0.007	0.113	0.133	0.003	-0.119	0.169	0.202	-0.039	-0.011	-0.084	0.000	0.047	0.218	-0.193	-0.217	-0.085
**HDL-C**	-0.001	-0.048	-0.033	-0.129	0.025	-0.092	-0.101	0.143	0.004	0.033	0.121	-0.003	0.118	0.037	0.054	0.078	-0.017	-0.185
**TG**	0.001	-0.028	0.030	0.014	-0.007	-0.079	0.050	0.172	0.055	0.046	0.002	-0.233	-0.111	-0.084	0.164	-0.127	-0.102	0.048
**LDL-c**	0.098	0.081	0.021	0.243	0.214	0.085	-0.095	0.086	0.229	-0.086	-0.002	-0.013	-0.038	0.130	0.173	-0.224	-0.108	-0.067
**Insulin B/I**	-0.016	-0.048	-0.137	-0.062	0.099	0.096	-0.005	0.083	-0.149	-.267*	0.085	-0.018	0.083	0.124	0.040	0.026	-0.206	0.083

*: $p<0.05$; **: $p<0.01$. BMI—Body mass index, BMI-SDS—standardized Body mass index, SBP—systolic blood pressure, DPB—diastolic blood pressure, HbA1c—glycated hemoglobin, TIR—Time in range; TC: Total cholesterol; HDL-C—High density lipoprotein cholesterol; TG—triglycerides; LDL-C—Low density lipoprotein cholesterol; Insulin B/I—treatment with B=1—basal bolus injections or I=2—intravenous infuser.

Table S4. Correlations between clinical characteristics and IPAQ-A items.

		n° sports lessons	minutes of walking during sports lessons	mins of sport during sports lessons	days vigorous physical activity during school	minutes of vigorous activity during school	days moderate physical activity during school	minutes of moderate activity during school	days of walking during school	minutes of walking during school	days of moderate activity at home	minutes of moderate activity at home	days travelling with motorized transportation	minutes travelling with motorized transport	days travelling using bike	minutes travelling using bike	days of travelling by foot	minutes travelling by foot	other days of vigorous activity	minutes of other days of vigorous activity	other days of moderate activity	minutes other days of moderate activity	other days walking	minutes other days walking	3,3 MET	4 MET	8 MET	total MET week
Gender	ρ Spearman	-0.015	0.032	0.132	0.181	0.181	0.000	0.005	0.127	0.127	-0.158	-0.213	-0.177	0.137	-0.045	-0.011	-0.089	-0.169	0.132	0.117	0.071	0.048	-0.035	-0.039	-0.121	0.078	0.141	0.117
	p-value	0.903	0.799	0.294	0.149	0.149	1.000	0.965	0.314	0.314	0.208	0.089	0.159	0.278	0.721	0.931	0.481	0.180	0.295	0.355	0.572	0.703	0.781	0.758	0.335	0.539	0.262	0.352
Age	ρ Spearman	-.403**	-.290*	-0.017	-0.199	-0.200	-.285*	-.285*	0.000	0.000	.254*	.328**	0.100	0.231	-0.001	-0.017	.362**	.326**	-0.098	-0.146	0.099	0.163	0.178	0.175	.391**	-0.051	-0.147	0.014
	p-value	0.001	0.019	0.895	0.111	0.110	0.021	0.022	1.000	1.000	0.041	0.008	0.427	0.064	0.996	0.893	0.003	0.008	0.438	0.245	0.434	0.194	0.157	0.163	0.001	0.687	0.242	0.914
Weight z-score	ρ Spearman	-0.207	-0.235	-.382**	-0.147	-0.148	0.022	0.016	-0.193	-0.193	.306*	0.102	0.058	-0.001	0.205	0.190	0.001	0.055	-0.100	-0.231	-0.029	-0.073	0.100	0.081	0.175	-0.174	-0.158	-0.133
	p-value	0.099	0.060	0.002	0.242	0.238	0.862	0.900	0.123	0.123	0.013	0.417	0.644	0.992	0.102	0.129	0.993	0.664	0.429	0.065	0.817	0.565	0.427	0.520	0.164	0.166	0.210	0.293
Height z-score	ρ Spearman	0.110	0.074	-0.142	-0.039	-0.039	-0.003	-0.006	-0.201	-0.201	0.154	-0.052	-0.024	-0.238	0.192	0.178	0.036	-0.120	0.116	-0.006	0.079	0.056	-0.199	-0.220	-0.058	-0.005	0.088	0.077
	p-value	0.385	0.564	0.264	0.760	0.759	0.979	0.960	0.111	0.111	0.226	0.681	0.848	0.059	0.128	0.158	0.777	0.347	0.363	0.965	0.535	0.662	0.114	0.081	0.650	0.969	0.489	0.545
BMI	ρ Spearman	-.353**	-.363**	-.398**	-0.199	-0.201	-0.060	-0.067	-0.113	-0.113	.291*	0.188	0.065	0.201	0.160	0.150	0.062	0.175	-0.199	-.296*	-0.019	-0.028	.254*	0.242	.312*	-0.185	-.264*	-0.191
	p-value	0.004	0.003	0.001	0.114	0.111	0.639	0.601	0.376	0.376	0.019	0.137	0.609	0.111	0.206	0.236	0.627	0.167	0.116	0.017	0.879	0.827	0.043	0.054	0.012	0.144	0.035	0.132
BMI-SDS	ρ Spearman	-.298*	-.314*	-.377**	-0.170	-0.172	0.018	0.011	-0.119	-0.119	.296*	0.148	0.076	0.125	0.181	0.170	0.009	0.131	-0.196	-.300*	-0.033	-0.066	0.217	0.208	.257*	-0.168	-.260*	-0.195
	p-value	0.017	0.012	0.002	0.179	0.174	0.886	0.932	0.348	0.348	0.018	0.244	0.550	0.325	0.153	0.180	0.944	0.301	0.121	0.016	0.796	0.602	0.086	0.099	0.040	0.185	0.038	0.122
BMI IOTF	ρ Spearman	-0.159	-.271*	-.270*	-0.221	-0.224	0.101	0.096	-0.052	-0.052	0.157	0.131	0.144	0.143	0.129	0.126	0.033	0.221	-0.184	-.302*	-0.053	-0.045	0.200	0.219	0.201	-0.076	-.249*	-0.152
	p-value	0.210	0.031	0.031	0.079	0.076	0.427	0.452	0.684	0.684	0.216	0.301	0.258	0.261	0.311	0.323	0.793	0.080	0.147	0.015	0.676	0.724	0.113	0.083	0.112	0.550	0.047	0.231
HbA1c	ρ Spearman	0.076	0.007	0.031	0.131	0.131	-0.087	-0.080	-0.083	-0.083	-0.153	-0.103	-0.079	-0.204	-.266*	-.276*	-0.047	-0.225	0.118	0.152	-0.023	0.010	0.009	-0.027	-0.233	0.009	0.160	0.147
	p-value	0.547	0.957	0.807	0.300	0.300	0.493	0.528	0.509	0.509	0.222	0.415	0.529	0.103	0.032	0.026	0.710	0.071	0.349	0.226	0.854	0.935	0.943	0.833	0.062	0.945	0.203	0.242
SBP	ρ Spearman	-0.058	-0.148	-0.008	-0.097	-0.096	-0.209	-0.209	-0.025	-0.025	0.038	0.063	.301*	0.207	-0.056	-0.067	.257*	0.172	0.004	-0.010	-0.049	0.001	0.118	0.158	0.165	-0.016	-0.002	-0.011
	p-value	0.654	0.250	0.954	0.451	0.456	0.104	0.104	0.846	0.846	0.769	0.627	0.017	0.106	0.668	0.604	0.043	0.183	0.977	0.935	0.703	0.997	0.360	0.219	0.201	0.902	0.985	0.929
DBP	ρ Spearman	-0.050	-0.052	0.046	0.026	0.026	-0.058	-0.058	0.171	0.171	0.023	0.122	.338**	0.090	0.031	0.010	.281*	0.192	-0.111	-0.228	-0.244	-.261*	0.004	0.048	0.139	-0.171	-0.157	-0.131
	p-value	0.701	0.690	0.722	0.841	0.841	0.653	0.653	0.184	0.184	0.860	0.345	0.007	0.488	0.809	0.941	0.027	0.135	0.391	0.075	0.056	0.041	0.973	0.711	0.282	0.184	0.223	0.312
Years from diagnosis	ρ Spearman	-.348**	-0.035	-0.024	0.192	0.192	-0.172	-0.175	-0.110	-0.110	.284*	0.120	-0.053	0.111	0.107	0.123	0.212	0.105	-0.002	-0.042	0.178	0.196	0.083	0.087	.262*	0.028	-0.027	0.164
	p-value	0.005	0.779	0.851	0.125	0.126	0.170	0.164	0.383	0.383	0.022	0.342	0.675	0.380	0.397	0.330	0.091	0.404	0.986	0.739	0.155	0.117	0.509	0.491	0.035	0.823	0.833	0.192
UI/Kg/die	ρ Spearman	0.092	0.122	-0.053	0.071	0.070	0.007	-0.002	0.100	0.100	-0.013	-0.143	-0.138	-0.151	0.019	0.027	0.078	0.076	0.136	0.113	-0.078	-0.019	-0.018	-0.021	-0.037	-0.003	0.121	-0.003
	p-value	0.464	0.332	0.675	0.573	0.580	0.958	0.988	0.428	0.428	0.919	0.255	0.274	0.230	0.880	0.833	0.535	0.545	0.280	0.369	0.538	0.879	0.886	0.866	0.770	0.980	0.337	0.979
Mean Blood Glucose	ρ Spearman	0.288	0.153	0.042							-0.144	-0.126	-0.225	-.373*	-0.300	-0.305	-0.171	-0.170	0.039	0.010	0.045	0.035	0.075	0.035	-0.222	0.046	0.062	0.151

	p-value	0.116	0.411	0.823							0.440	0.500	0.223	0.039	0.101	0.095	0.358	0.360	0.834	0.959	0.810	0.852	0.689	0.850	0.231	0.806	0.739	0.416
TIR %	ρ Spearman	-0.205	-0.086	0.009							0.175	0.255	0.173	.353*	0.320	0.328	0.158	0.238	-0.024	0.021	0.070	0.081	-0.067	-0.036	0.303	0.081	-0.042	0.001
	p-value	0.260	0.639	0.959							0.339	0.158	0.344	0.047	0.075	0.067	0.386	0.189	0.897	0.908	0.704	0.660	0.716	0.845	0.091	0.660	0.820	0.994
Total Cholesterol	ρ Spearman	0.017	-0.064	0.049	-0.176	-0.176	0.111	0.114	0.158	0.158	-0.006	0.000	-0.095	0.062	-0.020	0.006	-0.244	-0.120	-0.028	-0.019	0.115	0.107	0.154	0.147	-0.009	0.113	-0.042	0.037
	p-value	0.899	0.624	0.707	0.175	0.175	0.393	0.383	0.225	0.225	0.961	0.998	0.468	0.634	0.878	0.963	0.058	0.356	0.828	0.884	0.377	0.413	0.237	0.258	0.943	0.384	0.747	0.775
HDL Cholesterol	ρ Spearman	0.073	0.123	0.150	0.132	0.132	.266*	.265*	0.161	0.161	0.047	0.046	-0.087	0.103	0.080	0.097	-0.105	-0.060	0.033	0.033	0.113	0.059	-0.045	-0.023	-0.028	0.109	0.039	0.066
	p-value	0.572	0.341	0.245	0.305	0.305	0.037	0.037	0.211	0.211	0.715	0.720	0.504	0.424	0.536	0.454	0.415	0.643	0.800	0.799	0.383	0.647	0.726	0.860	0.830	0.398	0.766	0.611
Triglycerides	ρ Spearman	0.006	-0.183	-0.190	-0.129	-0.129	-0.154	-0.152	0.100	0.100	-0.074	-0.068	0.120	0.039	0.016	-0.001	-0.106	-0.044	-0.022	-0.100	-0.242	-0.200	.312*	.302*	0.017	-0.182	-0.048	-0.047
	p-value	0.964	0.155	0.138	0.318	0.318	0.233	0.237	0.438	0.438	0.566	0.598	0.355	0.761	0.900	0.994	0.412	0.736	0.865	0.441	0.058	0.119	0.014	0.017	0.897	0.156	0.710	0.719
LDL Cholesterol	ρ Spearman	-0.052	-0.093	-0.005	-0.188	-0.188	-0.041	-0.039	0.102	0.102	0.008	0.019	-0.078	0.007	-0.006	0.009	-0.182	-0.085	-0.034	-0.011	0.102	0.122	0.181	0.160	0.043	0.088	-0.046	0.023
	p-value	0.694	0.481	0.968	0.150	0.150	0.753	0.767	0.440	0.440	0.950	0.888	0.555	0.959	0.961	0.943	0.164	0.518	0.798	0.934	0.437	0.353	0.166	0.222	0.744	0.503	0.725	0.863
Insulin 1/2	ρ Spearman	0.014	0.011	0.187	-0.119	-0.119	-0.119	-0.119	-0.083	-0.083	-0.015	0.157	-0.016	0.179	0.018	0.046	0.073	0.097	0.024	-0.013	0.079	0.036	-0.125	-0.107	0.004	0.132	0.000	0.053
	p-value	0.913	0.931	0.135	0.346	0.346	0.346	0.346	0.509	0.509	0.907	0.212	0.898	0.155	0.888	0.717	0.566	0.443	0.849	0.921	0.529	0.777	0.321	0.394	0.972	0.294	1.000	0.673

*: $p<0.05$; **: $p<0.01$.

Table S5. Correlations between clinical characteristics and IPAQ-A items.

		n° sports lessons	minutes of walking during sports lessons	mins of sport during sports lessons	days vigorous physical activity during school	minutes of vigorous activity during school	days moderate physical activity during school	minutes of moderate activity during school	days of walking during school	minutes of walking during school	days of moderate activity at home	minutes of moderate activity at home	days travelling with motorized transportation	minutes travelling with motorized transport	days travelling using bike	minutes travelling using bike	days of travelling by foot	minutes travelling by foot	other days of vigorous activity	minutes of other days of vigorous activity	other days of moderate activity	minutes other days of moderate activity	other days walking	minutes other days walking	3,3 MET	4 MET	8 MET	total MET week
Gender	ρ Spearman	-0.015	0.032	0.132	0.181	0.181	0.000	0.005	0.127	0.127	-0.158	-0.213	-0.177	0.137	-0.045	-0.011	-0.089	-0.169	0.132	0.117	0.071	0.048	-0.035	-0.039	-0.121	0.078	0.141	0.117
	p-value	0.903	0.799	0.294	0.149	0.149	1.000	0.965	0.314	0.314	0.208	0.089	0.159	0.278	0.721	0.931	0.481	0.180	0.295	0.355	0.572	0.703	0.781	0.758	0.335	0.539	0.262	0.352
Age	ρ Spearman	-.403**	-.290*	-0.017	-0.199	-0.200	-.285*	-.285*	0.000	0.000	.254*	.328**	0.100	0.231	-0.001	-0.017	.362**	.326**	-0.098	-0.146	0.099	0.163	0.178	0.175	.391**	-0.051	-0.147	0.014
	p-value	0.001	0.019	0.895	0.111	0.110	0.021	0.022	1.000	1.000	0.041	0.008	0.427	0.064	0.996	0.893	0.003	0.008	0.438	0.245	0.434	0.194	0.157	0.163	0.001	0.687	0.242	0.914
Weight z-score	ρ Spearman	-0.207	-0.235	-.382**	-0.147	-0.148	0.022	0.016	-0.193	-0.193	.306*	0.102	0.058	-0.001	0.205	0.190	0.001	0.055	-0.100	-0.231	-0.029	-0.073	0.100	0.081	0.175	-0.174	-0.158	-0.133
	p-value	0.099	0.060	0.002	0.242	0.238	0.862	0.900	0.123	0.123	0.013	0.417	0.644	0.992	0.102	0.129	0.993	0.664	0.429	0.065	0.817	0.565	0.427	0.520	0.164	0.166	0.210	0.293
Height z-score	ρ Spearman	0.110	0.074	-0.142	-0.039	-0.039	-0.003	-0.006	-0.201	-0.201	0.154	-0.052	-0.024	-0.238	0.192	0.178	0.036	-0.120	0.116	-0.006	0.079	0.056	-0.199	-0.220	-0.058	-0.005	0.088	0.077
	p-value	0.385	0.564	0.264	0.760	0.759	0.979	0.960	0.111	0.111	0.226	0.681	0.848	0.059	0.128	0.158	0.777	0.347	0.363	0.965	0.535	0.662	0.114	0.081	0.650	0.969	0.489	0.545
BMI	ρ Spearman	-.353**	-.363**	-.398**	-0.199	-0.201	-0.060	-0.067	-0.113	-0.113	.291*	0.188	0.065	0.201	0.160	0.150	0.062	0.175	-0.199	-.296*	-0.019	-0.028	.254*	0.242	.312*	-0.185	-.264*	-0.191
	p-value	0.004	0.003	0.001	0.114	0.111	0.639	0.601	0.376	0.376	0.019	0.137	0.609	0.111	0.206	0.236	0.627	0.167	0.116	0.017	0.879	0.827	0.043	0.054	0.012	0.144	0.035	0.132
BMI-SDS	ρ Spearman	-.298*	-.314*	-.377**	-0.170	-0.172	0.018	0.011	-0.119	-0.119	.296*	0.148	0.076	0.125	0.181	0.170	0.009	0.131	-0.196	-.300*	-0.033	-0.066	0.217	0.208	.257*	-0.168	-.260*	-0.195
	p-value	0.017	0.012	0.002	0.179	0.174	0.886	0.932	0.348	0.348	0.018	0.244	0.550	0.325	0.153	0.180	0.944	0.301	0.121	0.016	0.796	0.602	0.086	0.099	0.040	0.185	0.038	0.122
BMI IOTF	ρ Spearman	-0.159	-.271*	-.270*	-0.221	-0.224	0.101	0.096	-0.052	-0.052	0.157	0.131	0.144	0.143	0.129	0.126	0.033	0.221	-0.184	-.302*	-0.053	-0.045	0.200	0.219	0.201	-0.076	-.249*	-0.152
	p-value	0.210	0.031	0.031	0.079	0.076	0.427	0.452	0.684	0.684	0.216	0.301	0.258	0.261	0.311	0.323	0.793	0.080	0.147	0.015	0.676	0.724	0.113	0.083	0.112	0.550	0.047	0.231
HbA1c	ρ Spearman	0.076	0.007	0.031	0.131	0.131	-0.087	-0.080	-0.083	-0.083	-0.153	-0.103	-0.079	-0.204	-.266*	-.276*	-0.047	-0.225	0.118	0.152	-0.023	0.010	0.009	-0.027	-0.233	0.009	0.160	0.147
	p-value	0.547	0.957	0.807	0.300	0.300	0.493	0.528	0.509	0.509	0.222	0.415	0.529	0.103	0.032	0.026	0.710	0.071	0.349	0.226	0.854	0.935	0.943	0.833	0.062	0.945	0.203	0.242
SBP	ρ Spearman	-0.058	-0.148	-0.008	-0.097	-0.096	-0.209	-0.209	-0.025	-0.025	0.038	0.063	.301*	0.207	-0.056	-0.067	.257*	0.172	0.004	-0.010	-0.049	0.001	0.118	0.158	0.165	-0.016	-0.002	-0.011
	p-value	0.654	0.250	0.954	0.451	0.456	0.104	0.104	0.846	0.846	0.769	0.627	0.017	0.106	0.668	0.604	0.043	0.183	0.977	0.935	0.703	0.997	0.360	0.219	0.201	0.902	0.985	0.929
DBP	ρ Spearman	-0.050	-0.052	0.046	0.026	0.026	-0.058	-0.058	0.171	0.171	0.023	0.122	.338**	0.090	0.031	0.010	.281*	0.192	-0.111	-0.228	-0.244	-.261*	0.004	0.048	0.139	-0.171	-0.157	-0.131
	p-value	0.701	0.690	0.722	0.841	0.841	0.653	0.653	0.184	0.184	0.860	0.345	0.007	0.488	0.809	0.941	0.027	0.135	0.391	0.075	0.056	0.041	0.973	0.711	0.282	0.184	0.223	0.312
Years from diagnosis	ρ Spearman	-.348**	-0.035	-0.024	0.192	0.192	-0.172	-0.175	-0.110	-0.110	.284*	0.120	-0.053	0.111	0.107	0.123	0.212	0.105	-0.002	-0.042	0.178	0.196	0.083	0.087	.262*	0.028	-0.027	0.164
	p-value	0.005	0.779	0.851	0.125	0.126	0.170	0.164	0.383	0.383	0.022	0.342	0.675	0.380	0.397	0.330	0.091	0.404	0.986	0.739	0.155	0.117	0.509	0.491	0.035	0.823	0.833	0.192
UI/Kg/die	ρ Spearman	0.092	0.122	-0.053	0.071	0.070	0.007	-0.002	0.100	0.100	-0.013	-0.143	-0.138	-0.151	0.019	0.027	0.078	0.076	0.136	0.113	-0.078	-0.019	-0.018	-0.021	-0.037	-0.003	0.121	-0.003
	p-value	0.464	0.332	0.675	0.573	0.580	0.958	0.988	0.428	0.428	0.919	0.255	0.274	0.230	0.880	0.833	0.535	0.545	0.280	0.369	0.538	0.879	0.886	0.866	0.770	0.980	0.337	0.979
Mean Blood Glucose	ρ Spearman	0.288	0.153	0.042							-0.144	-0.126	-0.225	-.373*	-0.300	-0.305	-0.171	-0.170	0.039	0.010	0.045	0.035	0.075	0.035	-0.222	0.046	0.062	0.151

	p-value	0.116	0.411	0.823							0.440	0.500	0.223	0.039	0.101	0.095	0.358	0.360	0.834	0.959	0.810	0.852	0.689	0.850	0.231	0.806	0.739	0.416
TIR %	ρ Spearman	-0.205	-0.086	0.009							0.175	0.255	0.173	.353*	0.320	0.328	0.158	0.238	-0.024	0.021	0.070	0.081	-0.067	-0.036	0.303	0.081	-0.042	0.001
	p-value	0.260	0.639	0.959							0.339	0.158	0.344	0.047	0.075	0.067	0.386	0.189	0.897	0.908	0.704	0.660	0.716	0.845	0.091	0.660	0.820	0.994
Total Cholesterol	ρ Spearman	0.017	-0.064	0.049	-0.176	-0.176	0.111	0.114	0.158	0.158	-0.006	0.000	-0.095	0.062	-0.020	0.006	-0.244	-0.120	-0.028	-0.019	0.115	0.107	0.154	0.147	-0.009	0.113	-0.042	0.037
	p-value	0.899	0.624	0.707	0.175	0.175	0.393	0.383	0.225	0.225	0.961	0.998	0.468	0.634	0.878	0.963	0.058	0.356	0.828	0.884	0.377	0.413	0.237	0.258	0.943	0.384	0.747	0.775
HDL Cholesterol	ρ Spearman	0.073	0.123	0.150	0.132	0.132	.266*	.265*	0.161	0.161	0.047	0.046	-0.087	0.103	0.080	0.097	-0.105	-0.060	0.033	0.033	0.113	0.059	-0.045	-0.023	-0.028	0.109	0.039	0.066
	p-value	0.572	0.341	0.245	0.305	0.305	0.037	0.037	0.211	0.211	0.715	0.720	0.504	0.424	0.536	0.454	0.415	0.643	0.800	0.799	0.383	0.647	0.726	0.860	0.830	0.398	0.766	0.611
Triglycerides	ρ Spearman	0.006	-0.183	-0.190	-0.129	-0.129	-0.154	-0.152	0.100	0.100	-0.074	-0.068	0.120	0.039	0.016	-0.001	-0.106	-0.044	-0.022	-0.100	-0.242	-0.200	.312*	.302*	0.017	-0.182	-0.048	-0.047
	p-value	0.964	0.155	0.138	0.318	0.318	0.233	0.237	0.438	0.438	0.566	0.598	0.355	0.761	0.900	0.994	0.412	0.736	0.865	0.441	0.058	0.119	0.014	0.017	0.897	0.156	0.710	0.719
LDL Cholesterol	ρ Spearman	-0.052	-0.093	-0.005	-0.188	-0.188	-0.041	-0.039	0.102	0.102	0.008	0.019	-0.078	0.007	-0.006	0.009	-0.182	-0.085	-0.034	-0.011	0.102	0.122	0.181	0.160	0.043	0.088	-0.046	0.023
	p-value	0.694	0.481	0.968	0.150	0.150	0.753	0.767	0.440	0.440	0.950	0.888	0.555	0.959	0.961	0.943	0.164	0.518	0.798	0.934	0.437	0.353	0.166	0.222	0.744	0.503	0.725	0.863
Insulin 1/2	ρ Spearman	0.014	0.011	0.187	-0.119	-0.119	-0.119	-0.119	-0.083	-0.083	-0.015	0.157	-0.016	0.179	0.018	0.046	0.073	0.097	0.024	-0.013	0.079	0.036	-0.125	-0.107	0.004	0.132	0.000	0.053
	p-value	0.913	0.931	0.135	0.346	0.346	0.346	0.346	0.509	0.509	0.907	0.212	0.898	0.155	0.888	0.717	0.566	0.443	0.849	0.921	0.529	0.777	0.321	0.394	0.972	0.294	1.000	0.673

*: $p<0.05$; **: $p<0.01$.
